# A management of a patient with chronic thromboembolic pulmonary hypertension prior to surgical resection for lung cancer

**DOI:** 10.1016/j.rmcr.2025.102269

**Published:** 2025-07-22

**Authors:** Satoshi Isomatsu, Kenichiro Takeda, Yu Shionoya, Toshihiko Sugiura, Shizu Miyata, Shun Imai, Jun Nagata, Yu Taniguchi, Akira Naito, Rika Suda, Ayako Shigeta, Nobuhiro Tanabe, Takuji Suzuki

**Affiliations:** a Department of Respirology, Graduate School of Medicine, Chiba University, Chiba, 260-8670, Japan; b Pulmonary Hypertension Center, Chibaken Saiseikai Narashino Hospital, Narashino, 275-0006, Japan; c Department of Cardiology, Graduate School of Medicine, Kobe University, Kobe, 650-0017, Japan

**Keywords:** Balloon pulmonary angioplasty, Chronic thromboembolic pulmonary hypertension, Lung cancer, Perioperative management

## Abstract

The basic treatment for chronic thromboembolic pulmonary hypertension (CTEPH) includes lifelong anticoagulant therapy and pulmonary endarterectomy; moreover, balloon pulmonary angioplasty (BPA) and vasodilators are also known to be effective. Surgery is the standard treatment for localized lung cancer. However, no established treatment guidelines exist for cases of coexisting CTEPH and lung cancer.

The patient was a 55-year-old woman who experienced dyspnea on exertion. She was diagnosed with non-small cell lung cancer, and surgery was scheduled; however, she was also diagnosed with CTEPH during preoperative examinations. She was referred to our hospital, where right heart catheterization revealed a mean pulmonary artery pressure (mPAP) of 39 mmHg. We prioritized the treatment of CTEPH, starting oral riociguat followed by BPA three times. The mPAP decreased to 27 mmHg. Then, right upper lobectomy and lymph node dissection were performed. After surgery, there was no significant worsening of right heart failure, and no recurrence of the lung cancer.

This case report presents a method for managing both CTEPH and primary lung cancer. The essence was the intensification of CTEPH treatment in anticipation of lobectomy.

## 1 Introduction

Patients with pulmonary hypertension (PH) are at high risk of perioperative complications and death [1]. Although several pulmonary circulatory markers are used for risk assessment [2,3], definitive protocol are not established.

The cornerstone of treatment for chronic thromboembolic pulmonary hypertension (CTEPH) is lifelong anticoagulation followed by pulmonary thromboendarterectomy (PEA) when feasible [2]. However, performing PEA is sometimes difficult when pulmonary artery thrombus are predominantly distributed in the peripheral regions [4]. Pulmonary vasodilators and ballon pulmonary angioplasty (BPA) have recently been recognized as important treatment options [2]. BPA has been reported to produce the same therapeutic outcomes as PEA [5]. In particular, additional treatment with BPA after oral administration of riociguat was shown to contribute to further improvements in exercise capacity, and pulmonary hemodynamics [6].

Here, we report a patient simultaneously diagnosed with CTEPH and operable lung cancer.

## 2 Case presentation

**Fig. 1:**
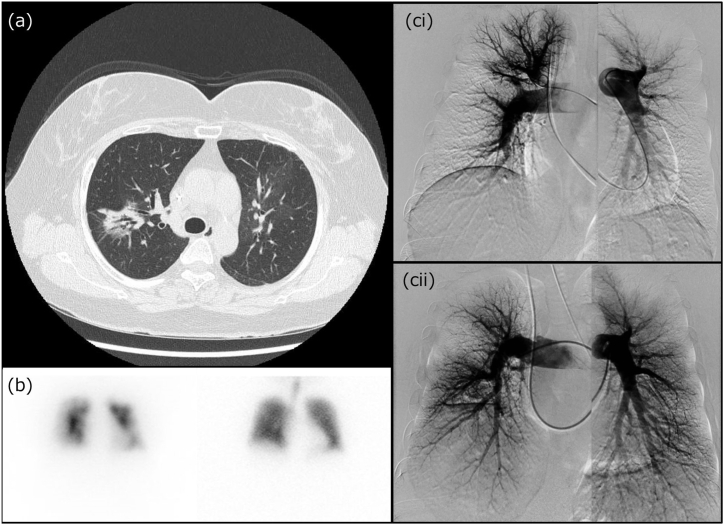
Radiological images (a) Chest computed tomography showing a part-solid ground-glass opacity with a long diameter of 3 cm in the upper lobe of the right lung (arrow), which turned out to be papillary adenocarcinoma. (b) Pulmonary perfusion scintigraphy presenting multiple defects bilaterally, whereas ventilation scintigraphy shows no defects. (ci) Pulmonary angiography performed at the time of diagnosis, showing weak distal perfusion on both sides. (cii) Pulmonary angiography performed after the third BPA session and lobectomy of the right upper lobe, showing an improvement in blood flow.

A 55-year-old woman, with a smoking history of 7.5 pack-years, had a medical history of hypertension, asthma, and left breast cancer, which was treated with surgery and postoperative chemotherapy. A central venous port was implanted in her chest. The patient had experienced exertional dyspnea for several years. During a health checkup, a tumor shadow was detected in the right middle lung field on chest radiography, leading to a referral to a nearby hospital. Chest computed tomography (CT) revealed a part-solid ground-glass opacity with a long diameter of 3 cm in the upper lobe of the right lung (Fig. 1a). No malignant findings were observed during bronchoscopy; however, the tumor did not shrink after six months of follow-up. The patient was referred for thoracic surgery for diagnostic treatment of the lung tumor, which was suspected to be primary lung cancer. However, preoperative electrocardiography showed increased p-waves and right-axis deviation, suggesting right heart overload. Echocardiography revealed right ventricular enlargement and the maximum velocity of tricuspid regurgitation (TRVmax) was approximately 3.9 m/s, suggesting pulmonary hypertension (PH). Additionally, pulmonary perfusion scintigraphy revealed multiple bilateral defects, whereas ventilation scintigraphy showed no defects (Fig. 1b). These results suggested chronic thromboembolic pulmonary hypertension (CTEPH), and treatment with edoxaban was initiated.

The patient was referred to our institution for further investigation. The values from right heart catheterization were as follows: pulmonary artery pressure (PAP; systolic/diastolic/mean), 69/18/39 mmHg; pulmonary artery wedge pressure (PAWP), 5 mmHg; right atrial pressure (RAP), 3 mmHg; pulmonary vascular resistance (PVR), 9.1 Wood units; cardiac output (CO), 3.72 L/min; cardiac index (CI), 2.24 L/min/m^2^ (Table 1). Blood test showed elevated level of brain natriuretic peptide (BNP); 216.1 pg/mL. Pulmonary arteriography revealed a peripherally predominant blood-flow defect, which led to a definitive diagnosis of CTEPH (Fig. 1ci). We considered that her pulmonary hemodynamics put her at high risk for requiring surgery for lung cancer. Therefore, the initial approach was to focus on the treatment of PH. After discussions with physicians, surgeons, and radiologists, a combination therapy with pulmonary vasodilators and balloon pulmonary angioplasty (BPA) was planned. After the introduction of riociguat, the patient underwent BPA three times. After the third BPA, mean PAP (mPAP) decreased to 27 mmHg and PVR decreased to 5.0 Wood units (Table 1). Pulmonary arteriography revealed improvement in peripheral blood flow, and BNP was reduced to 28.3 pg/mL.

**Table 1:** Findings of right heart catheter.

Parameters	At diagnosis	After the 3rd BPA	After the lobectomy	After 1 year of lobectomy
sPAP, mmHg	69	46	64	37
dPAP, mmHg	18	16	18	13
mPAP, mmHg	39	27	36	22
PAWP, mmHg	5	8	7	10
RAP, mmHg	3	NA	4	3
PVR, Wood unit	9.1	5.0	3.4	2.5
CO, L/min	3.72	3.80	8.57	5.84
CI, L/min/m^2^	2.24	2.22	5.04	3.33
PaO_2_, mmHg	65.4	NA	58.0	72.8
PvO_2_, mmHg	38.7	41.0	42.0	38.4

BPA, ballon pulmonary angioplasty; CI, cardiac index; CO, cardiac output; dPAP, diastolic pulmonary artery pressure; mPAP, mean pulmonary artery pressure; sPAP, systolic pulmonary artery pressure; PAWP, pulmonary artery wedge pressure; PaO_2_, partial pressure of arterial oxygen; PvO_2_, partial pressure of mixed venous oxygen; PVR, pulmonary vascular resistance; RAP, right atrial pressure.

Right upper lobectomy with lymph node dissection was performed in the thoracic surgery department. The pathological diagnosis after surgery was papillary adenocarcinoma T1cN1M0, pStage IIB (8th edition staging by the Union for International Cancer Control). Two weeks after surgery, the TRVmax surveyed by echocardiography was approximately 4.1 m/s, which was nearly equivalent to the value before BPA. However, the results of right heart catheterization performed at almost the same time indicated no exacerbation of PVR: PAP (systolic/diastolic/mean), 64/18/36 mmHg; PAWP, 7 mmHg; RAP, 4 mmHg; PVR, 3.4 Wood units; CO, 8.57 L/min; CI, 5.04 L/min/m^2^. Hypoxia due to lung resection was presumed to be the main cause of the increased cardiac output. Additionally, selexipag introduced postoperatively may have contributed to the increased cardiac output. The patient was discharged on long-term oxygen therapy because of persistent hypoxemia, which required 2 L/min oxygen inhalation. Since then, PH has stabilized, and the lung cancer has progressed without recurrence.

## 3 Discussion

This is the first report of successful management of a case of concomitant CTEPH and primary lung cancer. Although no study has suggested a direct relationship between CTEPH and lung cancer, it is generally recognized that a cancerous state increases coagulability and the risk of venous thromboembolism [7]. Additionally, in this case, the patient had a central venous port, which may have contributed to the development of CTEPH [8].

A recent study on perioperative outcomes in patients with PH found that 11.3 % had serious postoperative complications and 4.7 % died within 30 days after undergoing noncardiac surgery [9]. Bleeding is the serious perioperative complication in thoracic surgery. To the best of our knowledge, no reports have been made of pulmonary hypertension as a risk factor for intraoperative bleeding. However, anticoagulants and prostacyclin receptor agonists used to treat CTEPH will increase the risk of bleeding. Additionally, preoperative heparin administration was reported as the risk factor of bleeding requiring reoperation [10]. Fragility of pulmonary vessel walls due to pulmonary hypertension may also affect susceptibility to perioperative bleeding.

There are no firm indicators for evaluating postoperative risk [9]. Modifying some values, such as CI, RAP and BNP, which can be used for risk classification in PH, to low-risk range may help avoid perioperative adverse events [2,3].

However, in the management ahead of pulmonary resection for CTEPH patients, it is also necessary to thoroughly evaluate blood flow in each segment. In this case, the right heart catheterization performed after referral to our hospital showed high mPAP, PVR, and BNP, while CI was 2.2 L/min and RAP was 4 mmHg, both of which were not particularly unfavorable. However, pulmonary angiography and lung perfusion scintigraphy suggested that the blood flow around the tumor was well preserved; therefore, lobectomy for lung cancer was highly likely to exacerbate oxygenation and pulmonary hemodynamics. Therefore, it was important to improve perfusion in the remaining lung by implementing additional treatment preoperatively.

Adjuvant chemotherapy with cisplatin and vinorelbine is effective for completely resected stage II–III non-small cell lung cancer [11]. However, in this case, adjuvant chemotherapy was not administered because hydration from the cisplatin-based chemotherapy could worsen her pulmonary hemodynamics.

## 4 Conclusion

It was essential to administer appropriate medical therapy and BPA to make the patient tolerable for lung cancer surgery. But the treatment for cases of coexisting CTEPH and lung cancer remains unclear and further research is needed.

## CRediT authorship contribution statement

**Satoshi Isomatsu:** Writing – original draft, Investigation, Data curation. **Kenichiro Takeda:** Writing – review & editing, Project administration, Investigation, Data curation, Conceptualization. **Yu Shionoya:** Validation, Data curation. **Toshihiko Sugiura:** Validation, Supervision, Data curation. **Shizu Miyata:** Validation, Data curation. **Shun Imai:** Validation, Data curation. **Jun Nagata:** Validation, Data curation. **Yu Taniguchi:** Validation, Data curation. **Akira Naito:** Validation, Data curation. **Rika Suda:** Validation, Data curation. **Ayako Shigeta:** Validation, Data curation. **Nobuhiro Tanabe:** Validation, Data curation. **Takuji Suzuki:** Validation, Supervision.

## Patient consent

The patient provided written informed consent.

## Funding

None.

## Declaration of competing interest

The authors declare that they have no known competing financial interests or personal relationships that could have appeared to influence the work reported in this paper.
